# An Outbreak Threat Due to Chromobacterium violaceum Pseudobacteremia in a Tertiary Health Care Center: A Cross-Sectional Study

**DOI:** 10.7759/cureus.86603

**Published:** 2025-06-23

**Authors:** Udhaya Sankar Ranganathan, Radha Sugumaran, Nivetha Subramanian, Mangaiyarkarasi Thiyagarajan, Gopal Rangasamy

**Affiliations:** 1 Microbiology, Sri Manakula Vinayagar Medical College and Hospital, Puducherry, IND

**Keywords:** bacteremia, chromobacterium violaceum, infection control measures, investigation of outbreak, pseudo-outbreak

## Abstract

Background and Objective: *Chromobacterium violaceum*, a rare cause of sepsis, was isolated in high numbers from the blood cultures of patients over a short span of time. A nosocomial outbreak was suspected, and an epidemiological investigation was carried out to confirm the presence of an outbreak. The aim of the study is to determine whether the bloodstream infections are true clinical events or are due to sample contamination.

Methods: This cross-sectional study was done over a period of two months in 2024 at a tertiary health care center. A total of 21* C. violaceum *isolates were included. An outbreak investigation was started, and samples from different environmental niches of the suspected wards were collected to trace the source of the organism. Strain typing of* C. violaceum* was done by antibiogram typing, comparing their susceptibilities. All the data were entered in a Microsoft Excel sheet (Redmond, USA). The results are expressed as numbers and percentages.

Results: All the 21* C. violaceum* isolates had similar antibiotic susceptibility patterns. All the isolates were from blood cultures collected in the emergency room (ER). Collection of blood samples for culture in the ER was withheld, and an alternate nearby facility was used. Environmental samples from the ER did not yield growth of* C. violaceum,* but a water sample from a nearby construction site yielded* C. violaceum.* The patient’s case records were analyzed for evidence of sepsis. The patients are mostly males between the ages of 20 and 80 years.

Conclusion: Based on the circumstantial evidence, it is concluded that the threat is only a pseudo-outbreak.

## Introduction

*Chromobacterium violaceum *is an inhabitant of soil and water found in tropical and subtropical regions of the globe. Human infections due to this bacterium are rare but can cause life-threatening sepsis in immunocompromised hosts [1]. Severe infection in an immunocompetent host is usually rare, but a few cases with severe infections and fatal outcomes are reported in the literature [2,3]. The organism gains access to the host by entering through the non-intact skin and rarely through ingestion or inhalation. Wound abscess, local cellulitis, and regional lymphadenitis are the usual presentations, which may progress to widespread bacteremia and visceral abscess formation [4].

*C. violaceum* was isolated in unusual numbers from blood cultures in our laboratory over a short period of time. It is not very uncommon to have pseudo-bacteremia due to such uncommon organisms. There have been 72 documented clusters of pseudobacteremia between 1965 and 2010 [5]. By definition, a rise in the occurrence of a disease or organism above the baseline in a specific geographical area over a specific time period is called an outbreak, while an increase in the organisms isolated without evidence of infection is called a pseudo-outbreak. Pseudobacteremias are the common presentation of pseudo-outbreaks, while others like pseudomeningitis and pseudopneumonia are also reported in the literature [5]. Beyond the financial burden to the patient and the overuse of antimicrobials, pseudo outbreaks pose a serious risk of nosocomial infections if the source is not promptly identified and addressed.

Therefore, this study was conducted with the aim of determining whether the bloodstream infections are true clinical events or are due to sample contamination.

## Materials and methods

Study design and setting

This study is a cross-sectional study conducted by the Department of Microbiology in association with the Hospital Infection Control Committee (HICC) at a tertiary care teaching hospital in Puducherry, India. The study was done over a period of two months, from 8th March 2024 to 8th May 2024. The study was conducted following all the ethical guidelines and after getting approval from the institute’s ethics committee (EC/128/2024).

Study population

All patients whose blood culture yielded *C. violaceum*, irrespective of other demographic and clinical characteristics, were included in the study. The demographic and clinical data of the patients were retrieved from the electronic and physical patient registries.

Outbreak investigation

The outbreak investigation followed a systematic approach [6], starting with verifying the diagnosis and confirming the presence of an outbreak. The first isolate of *Chromobacterium violaceum* was reported on 8th March 2024, and subsequently, six additional isolates emerged between 22nd and 25th March 2024. Given the clustering of cases and the rarity of *C. violaceum* in the hospital's records, an outbreak was suspected, prompting an epidemiological investigation.

To track the outbreak, the Hospital Infection Control Committee (HICC) assessed patient locations, sample collection sites, and ward transfers while listing possible risk factors such as contamination of blood culture vials, culture media, disinfectants, intravenous fluids, and the environment, including tap water. To determine contamination sources, five sterile blood culture bottles from the outbreak locations were incubated for sterility checks. Environmental samples were collected from eight sites, including bed railings, vital monitors, IV pumps, patient trolleys, bedside tables, nursing stations, sinks, and water taps, using saline-moistened sterile cotton swabs. One swab from each site was collected consecutively for three days. The swabs were suspended in brain heart infusion broth (BHI) incubated at 37°C for 48 hours and then subcultured to 5% sheep blood agar (SBA). In-use cotton pledgets and dressing material were also collected, and bits of them were inoculated on 5% SBA. In-use antiseptics, intravenous fluids, and tap water were collected, and 1 mL was inoculated in 5 mL of BHI broth. After incubating at 37°C for 48 hours, subcultures were made to 5% SBA. Any growth on 5% SBA after 48 hours of incubation at 37°C was picked up and subjected to identification by Vitek2. One plain BHI tube and one BHI tube with ATCC *Escherichia coli* 25922 were used as sterility control and growth control, respectively.

**Figure 1:**
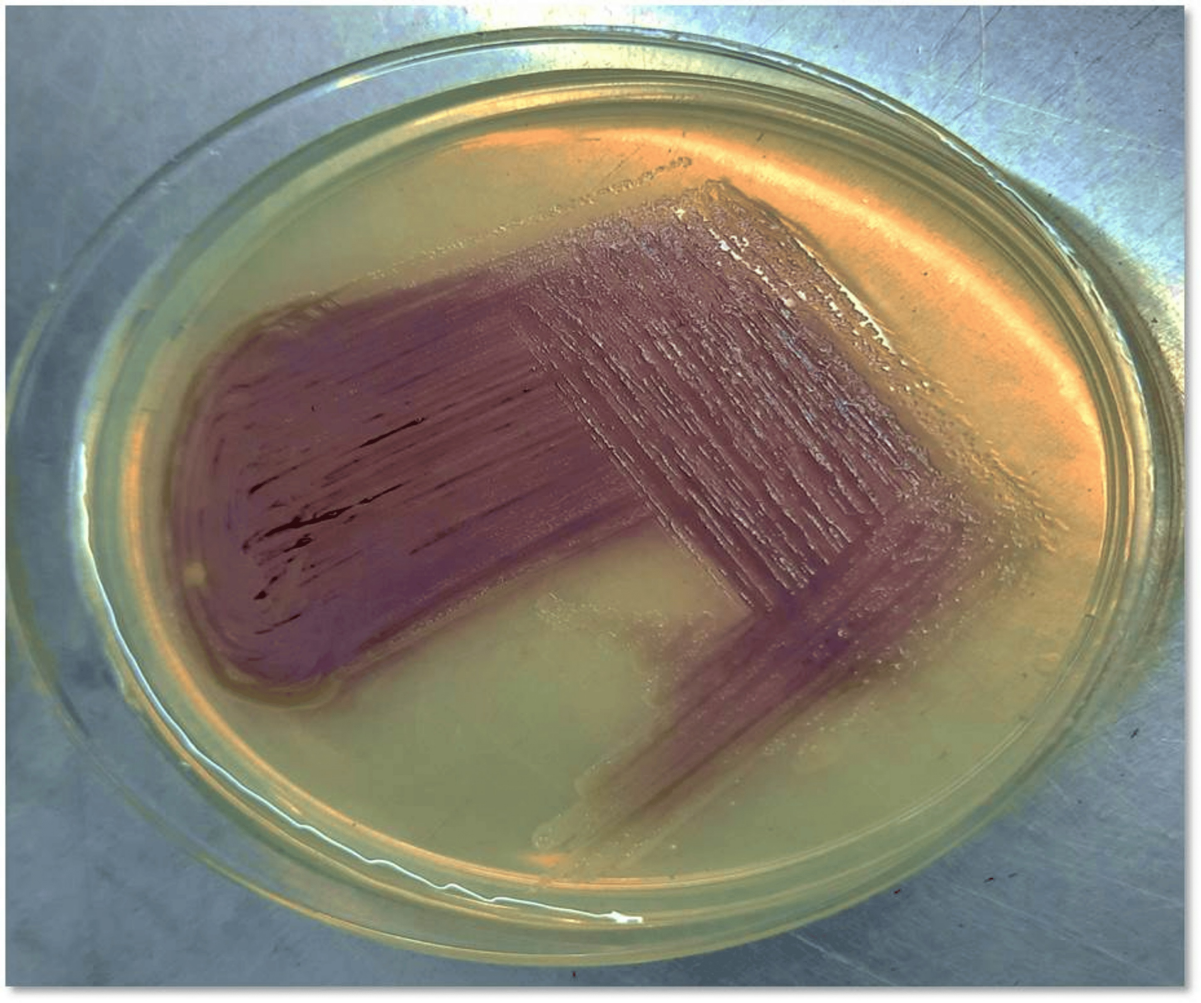
Violet-colored colonies of C. violaceum on nutrient agar

The outbreak was defined as all patients exhibiting clinical signs of sepsis with *C. violaceum* isolated from their blood samples. Blood culture was done using BACTEC FX40 automated blood culture systems, followed by subculturing on 5% SBA and MacConkey agar. The presence of characteristic violet-colored colonies suggested *C. violaceum* (Figure 1).

Conventional biochemical tests showed catalase and oxidase positivity, while other biochemical reactions supported the identification. The relatedness of the isolates was determined by comparing the antimicrobial susceptibilities of the organism [7]. Antimicrobial susceptibility testing (AST) was done using the Kirby-Bauer disk diffusion method with antibiotic disks from Himedia Labs, Mumbai, India. Since *C. violaceum* lacks specific disk diffusion breakpoints in Clinical Laboratory Standards Institute (M100, 2024) guidelines, zone diameters were interpreted based on *Pseudomonas aeruginosa* breakpoints [8]. Further confirmation of identification and AST was done using Vitek 2.

Antibiogram typing was done to determine the clonality by comparing the susceptibilities of the isolates to the antibiotics amikacin, gentamicin, ciprofloxacin, meropenem, imipenem, and co-trimoxazole. Isolates having identical susceptibility and/or resistance patterns for all the antibiotics tested were considered as matching isolates in this study. While a molecular typing method like pulse-field gel electrophoresis (PFGE) or whole-genome sequencing could have given a better clonal matching, antibiogram typing was preferred in this study due to the resource-poor setting, cost restrictions, and the practicality of the situation demanding urgent intervention. The investigation assessed facility structures, engaged with involved staff, implemented infection control measures, communicated findings to stakeholders, and evaluated the impact of interventions. Through systematic hypothesis testing and epidemiologic analyses, the outbreak management team aimed to pinpoint common links and effectively contain the spread of infection.

Statistical analysis

All the data were entered into a Microsoft Excel sheet. The results are expressed as numbers and percentages. Given the small study population, we employed descriptive statistics to summarize key variables and explore possible patterns or associations.

## Results

**Figure 2:**
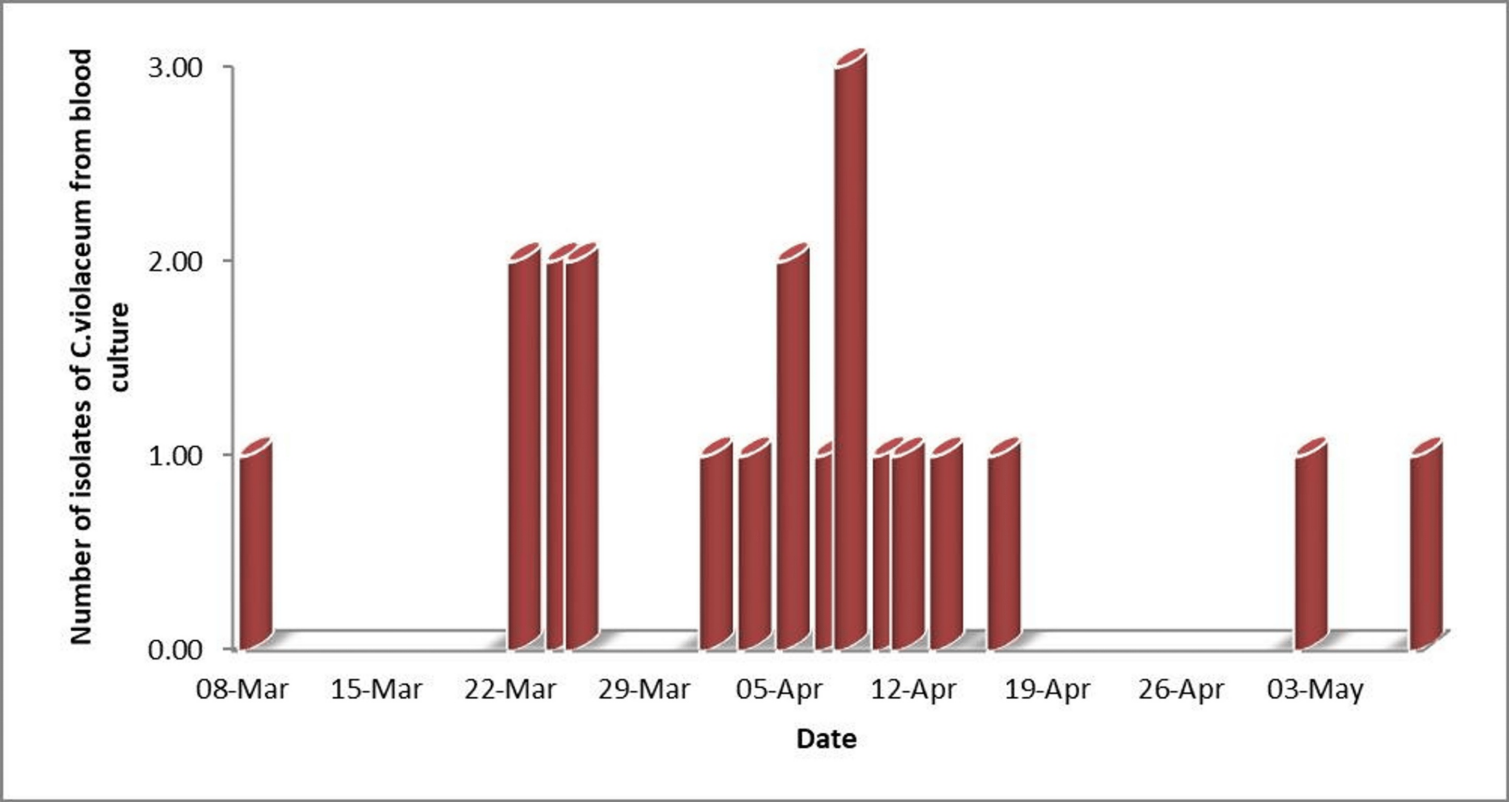
Time frame of C. violaceum isolated in blood culture Image is created by the author

A total of 21 *C. violaceum* were isolated from blood samples from different patients over a period of two months (Figure 2).

In all cases, the emergency room (ER) was identified as the common source of blood collection for blood culture (Table 1).

**Table 1:** Location history of patient

Current location of the patient	Transfer from	Location when Blood culture specimen was collected	Total number of patients
Intensive care unit	Emergency room	Emergency room	5
General medicine ward	Emergency room	Emergency room	14
General surgery ward	Emergency room	Emergency room	1
Orthopedics ward	Emergency room	Emergency room	1

The majority of the isolates were from male patients; the male-to-female ratio was 6:1 (Table 2).

**Table 2:** Age and gender distribution of patients in the emergency room (ER) from whom C. violaceum was isolated

Age Group	Male	Female	Total
21-30	2	0	2
31-40	2	1	3
41-50	4	1	5
51-60	5	1	6
61-70	3	0	3
71-80	2	0	2
Total	18	3	21

Since there was a clustering of isolates from blood cultures collected from the ER, it was identified as the source of the outbreak. On evaluation by the HICC team, two patients were diagnosed with urinary tract infection with significant growth of *Escherichia coli* in urine culture, two patients were diagnosed with laboratory-confirmed dengue, and one patient was diagnosed with acute pancreatitis. The remaining patients had an undifferentiated fever for a short duration, and they recovered uneventfully. Repeat blood cultures from the patients after transfer did not grow *C. violaceum*. The absence of infection symptoms and negative cultures following relocation suggests that the initial isolates were likely contaminants.

The environmental samples collected from various locations of the ER did not grow *C. violaceum*. The sterile blood culture bottles retrieved from the ER for sterility check also did not flag positive even after seven days of incubation, ruling out media contamination. However, all 21 isolates had similar antibiotic susceptibility patterns, indicating all the strains had similar phenotypes. High strain similarity was observed, with resistance profiles including amikacin and gentamicin, while remaining sensitive to cotrimoxazole, ciprofloxacin, meropenem, and imipenem. It was observed that civil construction work was in progress adjacent to the ER. A water sample collected from the construction area yielded the same antibiogram phenotype of *C. violaceum*.

From all these findings, it was concluded that *C. violaceum* could have been most probably introduced as contamination during venipuncture, but the exact vehicle of transmission remained unidentified. To prevent pseudobacteremia from turning into an outbreak, infection prevention measures were implemented. The first step was stopping the collection of blood samples for culture in the ER, and an alternate nearby facility was used for the same. The hospital floorings were thoroughly washed, and all touch areas were meticulously disinfected. The in-use disinfectants, antiseptics, and cotton pledgets were replaced with a new lot. Since the civil construction work adjacent to the ER grew *C. violaceum*, it was sealed from the ER side. Following the interventions, there were no further occurrences of *C. violaceum pseudobacteremia* from the ER room.

## Discussion

Pseudobacteremia can occur due to blood culture bottle contamination at the manufacturer level during the venipuncture or during the handling of samples in the laboratories. During venipuncture, contaminated cotton pledgets and contaminated antiseptics used for disinfecting the venipuncture site can be the source of contamination. Koo HS et al. found contaminated cotton pledgets to be the source of a pseudooutbreak due to *Pantoea* species in their study [9]. A systematic review by Yoon et al. showed contaminated disinfectants to be the cause of bacteremia due to *Achromobacter* species in seven studies included in their review [10]. Song JE et al. found contaminated chlorhexidine to be the cause of *Burkholderia cepacia pseudobacteraemia *in their study [11]. In our study, the blood culture media and analyzers were not contaminated. The cotton pledgets and antiseptics used for venipuncture did not yield any growth either. Intravenous infusates are well-known causes of nosocomial bacteremia [12]. In our study, commonly used intravenous fluids in the ER were sampled, and they did not grow any bacteria.

In a study by Cambel et al., construction work was found to be a source of clustering of *Bacillus *species in blood cultures [13]. In our study, the ER had a construction project in progress, and water samples from the site yielded growth of *C. violaceum*. Unfortunately, the source of *C. violaceum* contributing to the contamination of blood cultures could not be identified, as none of the environmental samples from the ER yielded *C. violaceum*. Nevertheless, we strongly suspected the construction work to be the source of the organism.

*C. violaceum* can cause life-threatening infections, particularly in immuno-compromised patients. Considering this fact, various infection control measures were implemented in the ER room even though the exact source of contamination remained unidentified. Following the interventions, there were no further occurrences of *C. violaceum* pseudo-outbreaks. Since the outbreak was limited only to a single location of the hospital (ER), the infection control measures were successful in preventing the spread of the outbreak. Therefore, timely deployment of infection control measures may prevent a serious nosocomial epidemic, even in cases when the precise source of the outbreak cannot be determined, with the help of circumstantial evidence.

Numerous pseudo-outbreaks attributed to bacteria, fungi, and viruses have been documented in the literature [11-16]. To the best of our knowledge, this is the first instance of *C. violaceum* linked to a nosocomial pseudobacteremia outbreak.

One limitation of the study is its inability to conclusively determine the outbreak's source. The slightly delayed investigation may have resulted in potential sources being cleaned or altered, erasing vital evidence. This highlights the need for timely outbreak investigations, especially considering the significant gap since the last positive isolate. Additionally, the contamination may have been intermittent, further complicating detection during the sampling period. While antibiogram typing was used for strain typing in our study, a molecular typing method would have greatly enhanced reliability.

## Conclusions

The spatial clustering of cases, the absence of the pathogen in follow-up samples after patient relocation, and the identification of alternate fever etiologies collectively indicate that the event constitutes a pseudo-outbreak rather than a true outbreak. Nevertheless, the unidentified environmental source leaves some degree of uncertainty to the conclusion. Ultimately, this underscores the critical importance of promptly recognizing an outbreak and initiating a timely investigation to accurately identify its source. Clinical microbiologists should be cautious when reporting uncommon pathogens and promptly alert the infection control team upon detecting clustered isolates.
